# Enzymatic Production of 3-OH Phlorizin, a Possible Bioactive Polyphenol from Apples, by *Bacillus megaterium* CYP102A1 via Regioselective Hydroxylation

**DOI:** 10.3390/antiox10081327

**Published:** 2021-08-23

**Authors:** Ngoc Anh Nguyen, Ngoc Tan Cao, Thi Huong Ha Nguyen, Jung-Hwan Ji, Gun Su Cha, Hyung-Sik Kang, Chul-Ho Yun

**Affiliations:** 1 School of Biological Sciences and Biotechnology, Graduate School, Chonnam National University, Yongbong-ro 77, Gwangju 61186, Korea; nguyenanh@jnu.ac.kr (N.A.N.); 187529@jnu.ac.kr (N.T.C.); huongha0207@gmail.com (T.H.H.N.); 216704@jnu.ac.kr (J.-H.J.); kanghs@jnu.ac.kr (H.-S.K.); 2 Namhae Garlic Research Institute, 2465-8 Namhaedaero, Gyeongsangnamdo 52430, Korea; gscha450@gmail.com; 3 School of Biological Sciences and Technology, Chonnam National University, Yongbong-ro 77, Gwangju 61186, Korea

**Keywords:** bacterial CYP102A1, dihydrochalcone, glucoside, regioselective hydroxylation, phloretin, phlorizin

## Abstract

Phlorizin is the most abundant glucoside of phloretin from the apple tree and its products. Phlorizin and its aglycone phloretin are currently considered health-beneficial polyphenols from apples useful in treating hyperglycemia and obesity. Recently, we showed that phloretin could be regioselectively hydroxylated to make 3-OH phloretin by *Bacillus megaterium* CYP102A1 and human P450 enzymes. The 3-OH phloretin has a potent inhibitory effect on differentiating 3T3-L1 preadipocytes into adipocytes and lipid accumulation. The glucoside of 3-OH phloretin would be a promising agent with increased bioavailability and water solubility compared with its aglycone. However, procedures to make 3-OH phlorizin, a glucoside of 3-OH phloretin, using chemical methods, are not currently available. Here, a biocatalytic strategy for the efficient synthesis of a possibly valuable hydroxylated product, 3-OH phlorizin, was developed via CYP102A1-catalyzed regioselective hydroxylation. The production of 3-OH phlorizin by CYP102A1 was confirmed by HPLC and LC–MS spectroscopy in addition to enzymatic removal of its glucose moiety for comparison to 3-OH phloretin. Taken together, in this study, we found a panel of mutants from *B. megaterium* CYP102A1 could catalyze regioselective hydroxylation of phlorizin to produce 3-OH phlorizin, a catechol product.

## 1. Introduction

Apples are one of the most important dietary sources of polyphenols [1]. They have many benefits to human health; they are also cheap and can be found all over the world [2,3,4,5]. As an agricultural activity, processing of apples produces approximately 25% of the apple pomace such as skin, stem, flesh, and seeds [2]. As the apple pomace has diverse adverse effects on the environment, use of apple pomace as a target for valorization maybe of interest to exploit the waste. In the last few years, the search for inexpensive and renewable sources of polyphenol compounds has been attracting researchers’ interest [6,7,8]. Interestingly, apples contain over 60 different phenolic compounds [9]. Five major groups of polyphenols are found in apples: flavonols, flavanols, phenolic acids, anthocyanins, and dihydrochalcones [10,11,12].

**Figure 1:**
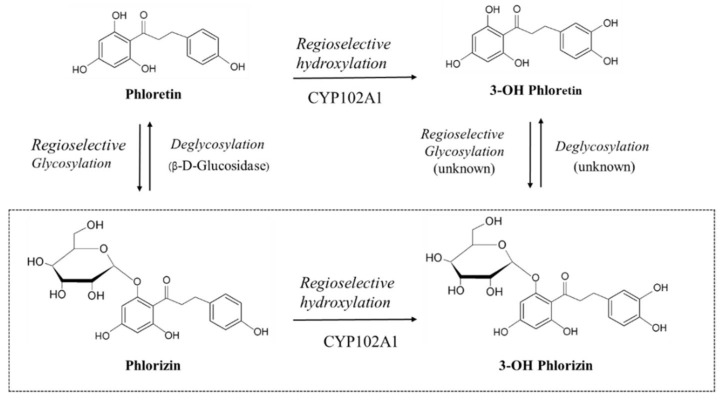
CYP102A1 catalyzed regioselective hydroxylation of phlorizin to produce 3-OH phlorizin. A scheme of currently found enzymatic interconversion reactions of phloretin, 3-OH phloretin, and phlorizin is also shown. In the present study, enzymatic conversion from phlorizin to 3-OH phlorizin was studied using CYP102A1 in the presence of NADPH.

Up to now, over 200 different dihydrochalcone (DC) compounds have been found in over 30 plant families [13]. Although phlorizin was thought to exist only in apples for a long time, now we know that it is also present in other plant species, such as *Lithocarpus* [14], cranberry [15], strawberry [16], and rose [17]. Among the DCs, phlorizin (phloretin 2′-O-glucoside) is the most popular and widely studied (Figure 1). It is a natural polyphenol glycoside, which was first isolated from the apple tree bark [18]. Long-term use of phlorizin can decrease blood glucose level and reduce lipid metabolism in diabetic mice [19]. This polyphenol was found to be the specific and competitive inhibitor of sodium–glucose symporters (SGLTs) in the small intestine (SGLT1) and the kidney (SGLT2); hence, phlorizin may improve hyperglycemia by blocking renal glucose reabsorption and intestinal glucose absorption. Phlorizin can also increase insulin sensitivity by changing the structure of gut microbiota in obese mice having type 2 diabetes [20]. In addition to its antioxidant activity [21], it can antagonize the induction of glycogen synthase kinase-3β and the β-catenin phosphorylation in osteoblasts and aged mouse bones [22]. Overall, phlorizin has high potential benefits for human health, including pharmaceutical application.

Three-hydroxyphlorizin (3-OH phlorizin) is a natural glycoside directly produced from natural sources, mainly found in Malus plants, and it can be purified by the solvent extraction method [23,24]. It has been reported that the concentration of 3-OH phlorizin is approximately 0.11 mg in 100 g *Malus domestica* [9,25,26]. However, it is not commercially available now because of its low abundance in natural sources. At present, its physiological and pharmacological activity has not been extensively studied. Furthermore, no methods for chemical synthesis of 3-OH phlorizin have been reported. Because the selective and efficient syntheses of 3-OH phlorizin and its derivatives by traditional chemical methods are difficult, its production via biocatalysis should be a useful alternative. Recently, we showed that phloretin, an aglycone of phlorizin, could be regioselectively hydroxylated to make 3-OH phloretin by *Bacillus megaterium* CYP102A1 [27] and human P450 enzymes [28]. Interestingly, the 3-OH phloretin has a potent inhibitory effect on differentiating 3T3-L1 preadipocytes into adipocytes and lipid accumulation [27].

Cytochrome P450 (P450 or CYP) is ubiquitous in nature [29]. To date, more than 350,000 CYP genes have been found in living organisms [30]. They can catalyze diverse metabolic oxidation reactions of exogenous and endogenous compounds [31]. The catalytic diversity and vast substrate range of P450s have great potential for many biotechnological applications, including the biocatalytic synthesis of drug metabolites, fine chemicals, steroids, lipids, and natural products [32,33,34,35]. CYP102A1 (P450 BM3) from B. megaterium is catalytically self-sufficient because it contains both a heme domain and a diflavin reductase domain responsible for the substrate oxidation and the electron transfer to the heme domain, respectively. A large set of CYP102A1 mutants have been generated through rational design and random mutagenesis to engineer enzymes with higher activity and stability [32,34,36,37]. Now, it is generally accepted that engineered CYP102A1 can be used to synthesize a wide variety of human metabolites of drugs and steroids, as well as other industrially important fine chemicals.

The major goal of this study was to find a biocatalytic strategy for the production of a monohydroxylated product at C-3 from phlorizin. A panel of CYP102A1 mutants were used for the efficient synthesis of a possible bioactive product from phlorizin. To our knowledge, the enzymatic hydroxylation of phlorizin has not been reported yet. Here, we report for the first time on biocatalytic synthesis of 3-OH phlorizin, a major product of phlorizin (Figure 1).

## 2. Materials and Methods

### 2.1. Materials

Glucose-6-phosphate, glucose-6-phosphate dehydrogenase, β-glucosidase from almonds, β-nicotinamide adenine dinucleotide phosphate (NADP^+^), and phlorizin were purchased from Sigma-Aldrich (St. Louis, MO, USA). All of the other chemicals used here were of the highest grade commercially available.

### 2.2. CYP102A1 Mutants to Screen Highly Active Phlorizin Hydroxylases

A large set of mutant collections of CYP102A1 was gathered from our previous work to make human metabolites of natural products and drugs by using various CYP102A1 mutants [6,7,38]. The wild type (WT) CYP102A1 from *B. megaterium* [39] did not show activities of phlorizin hydroxylation. Mutants from M1 to M17 have mutations at the amino acids located in the substrate channel and active site [40]. Twenty-six mutants from B1 to M850 were selected from approximately 500 blue colonies obtained as described previously [39] (Supplementary Table S1).

### 2.3. Construction of an Expression Plasmid for the CYP102A1 Gene

Error-prone polymerase chain reaction (EP-PCR) was performed on the CYP102A1 heme domain (1-430 amino acid residues) of M16V2 to make more active mutants exhibiting phlorizin hydroxylation activity. The M16V2, a chimeric protein, was generated by exchanging the reductase domain of mutant M16 with that of the natural variant V2 [39,41]. The heme domain-coding gene was amplified by the PCR with oligonucleotide primers BamHI/SacI restriction sites: BamHI forward, 5′-ataGGATCCatgacaattaaagaaatg cctc-3′ and SacI reverse, 5′-ataGAGCTCgtagtttgtatgatcttcaaagtcaaag tg-3′. Libraries of random mutants were constructed using a Diversify^@^ PCR Random Mutagenesis Kit (Tanaka, Clontech, CA, USA). The reaction mixture (V_T_ = 50 μL) contained 0.2 μM of each primer, 0.2 mM dNTP (0.05 mM each of dATP, dGTP, dCTP, and dTTP), Taq DNA polymerase (5 units/µL), MgCl_2_ (2.5 or 5 mM), and MnCl_2_ (0.1 or 0.15 mM) in 10 mM Tris-HCl containing 50 mM KCl (pH 8.4, 25 °C). The mutation rate was 2.9 mutations per 1290 bp. The library size of the screened mutants was approximately 1.0 × 10^6^.

The randomized plasmid library was first screened using a blue color colony-based method, as described previously [6]. Approximately 500 blue-colored colonies were transferred into 96-well deep well plates, containing 0.3 mL of Luria–Bertani (LB) media with ampicillin (100 μg/mL) at 37 °C and 170 rpm overnight. The aliquots of cell culture (1%, v/v) were inoculated in 0.3 mL of Terrific Broth (TB) medium containing 100 μg/mL ampicillin, 1 mM thiamine, trace elements, 50 μM FeCl_3_, 1 mM MgCl_2_, and 2.5 mM (NH_4_)_2_SO_4_. The whole-cell activity assay of the CYP102A1 mutants was performed using 4-nitrophenol as a substrate.

### 2.4. Expression of CYP102A1 Mutants

The plasmid of WT and a panel of CYP102A1 mutants were expressed in the *Escherichia coli* strain DH5αF’–IQ cells. The cultures (5 mL) were grown overnight in LB broth with ampicillin (100 μg/mL) selection at 37 °C. Pre-culture cells were used to inoculate ampicillin, 1.0 mM thiamine, 1 mM MgCl_2_, 50 μM FeCl_3_, 2.5 mM (NH_4_)_2_SO_4_, and trace elements. Cells were grown at 37 °C and 190 rpm to an OD of between 0.6 and 0.8. After protein expression was induced by the addition of 0.5 mM isopropyl-ß-D-thiogalactopyranoside with δ-aminolevulinic acid (1.0 mM). After 22 h, cells were harvested using centrifugation (15 min, 3500 rpm, 5 °C). The cell pellet was resuspended in Tris-acetate buffer (50 mM, pH 7.6) containing 250 mM sucrose and 0.25 mM ethylenediaminetetraacetic acid, and lysed by sonication. To remove cell debris, the lysate was centrifuged at 100,000× *g* (90 min, 4 °C) to collect the soluble fraction. The cytosolic (supernatant) fraction was stored at −80 °C until use. The CO-difference spectrum with ε = 91 mM^−1^ cm^−1^ was used to measure the CYP102A1 concentration [42].

### 2.5. Hydroxylation of Phlorizin Catalyzed by CYP102A1 Mutants

Typical steady-state reactions for the oxidation of phlorizin were carried out in 0.25 mL of 100 mM potassium phosphate buffer (pH 7.4) for 30 min at 37 °C. The reaction mixtures contained WT or mutant enzymes (0.4 μM), phlorizin (0.2 mM), and an NADPH regeneration system (final concentrations: 10 mM glucose-6-phosphate, 0.5 mM NADP^+^, and 1.0 IU yeast glucose-6-phosphate dehydrogenase/mL). The reaction was terminated by adding 600 μL ice-cold ethyl acetate. After the centrifugation of the reaction mixture (3000× *g* for 20 min), a 0.3 mL aliquot of the ethyl acetate layer (upper phase) was transferred to a clean glass tube. The ethyl acetate was removed under a N_2_ stream.

Phlorizin and its products were analyzed by HPLC using a Gemini C18 column (4.6 × 150 mm, 5 μm, 110 Å; Phenomenex, Torrance, CA, USA) with the mobile phase A (water containing 0.1% (*v*/*v*) formic acid and 0.5% (*v*/*v*) methanol) and the mobile phase B (acetonitrile only) [39]. Product formation was analyzed at A_285_ with the flow rate of the elution solvent at 1.0 mL/min by a gradient pump (LC-20AD; Shimadzu, Kyoto, Japan) with the following gradient: 0–3 min maintained at 9% (*v/v*) mobile phase B; 3–20 min gradually increased, reaching 30% (*v*/*v*) mobile phase B; 20–21 min decreased to 9% (*v*/*v*) mobile phase B; and 21–30 min maintained at 9% (*v*/*v*) mobile phase B.

To determine the kinetic parameters of phlorizin 3-hydroxylation by CYP102A1 mutants, 0.01–1 mM phlorizin, 0.40 μM enzymes, and an NADPH-generating system were used. After the reaction mixture was incubated at 37 °C for 60 min, the product formation rate was determined by HPLC, as described above. A stock of substrate solution was prepared in ethanol and diluted in the enzymatic reactions to the final organic solvent concentration of <1% (*v*/*v*). The experimental results were determined using nonlinear regression analysis using GraphPad Prism program (GraphPad Software, San Diego, CA, USA). The data were fit to the standard Michaelis-Menten equation: *v* = *k_cat_* [E][S]/([S] + *K_m_*), where the velocity (*v*) of the reaction is a function of the rate-limiting step in turnover (*k_cat_*), the enzyme concentration ([E]), substrate concentration ([S]), and the Michaelis constant (*K_m_*).

The time course analysis of phlorizin hydroxylation was determined with 200 μM phlorizin and 0.40 μM enzymes in potassium phosphate buffer (100 mM, pH 7.4). The reaction was initiated by adding the NADPH-generating system and incubated for 10, 30, 60, 90, 120, and 150 min at 37 °C. Product formation was analyzed using HPLC, as described above.

### 2.6. LC–MS Analysis

To identify the products of phlorizin produced by the CYP102A1 mutants, LC–MS analysis of the products was performed for comparison of the LC profile and MS fragmentation pattern with those of the authentic compound: phlorizin. The reaction residue was solubilized with 100 μL of mobile phase by vortex mixing. An aliquot (5 μL) of this solution was injected onto the LC column. LC–MS analysis was carried out on a Thermo Scientific Accela^TM^, Xcalibur^TM^ 3.0, and TSQ Quantum^TM^ Access MAX system with the heated electrospray ionization interface with a HESI II probe (Thermo Fisher Scientific, Waltham, MA, USA). The separation was done on a ZorBax SB-C18 column (4.6 × 250 mm, 5 μm, 80 Å; Agilent Technologies, Santa Clara, CA, USA); the gradient of mobile phase was performed with 0.1% formic acid (*v*/*v*) and 0.5% (*v*/*v*) methanol in water (A) in acetonitrile (B), and the mobile phase was delivered at a flow rate of 1.0 mL/min.

The initial composition of mobile phase B was 9% (*v*/*v*) after 11 min; the mobile phase B composition increased to 15% (*v*/*v*) over 17 min, increased to 17% (*v*/*v*) over 7 min, increased to 60% (*v*/*v*) over 17 min, increased to 100% over 1 min, and finally re-equilibrated to the initial conditions over 10 min. Thus, the total run time for each sample was 35 min. The temperatures of the column and autosampler were maintained at 30 °C and 4 °C, respectively. The mass spectra were recorded by electrospray ionization in negative mode to identify the phlorizin products. Full scan mass spectrometry was used. The collision energy and scan rate were 20 V and 0.5 spectral/s, respectively. The temperature of both the vaporizer and capillary was 320 °C. Nitrogen sheath gas pressure and auxiliary gas pressure were 60 psi and 20 psi, respectively, with a spray voltage of 3000 V.

### 2.7. Spectral Binding Titration

All spectrophotometric measurements were performed using a Shimadzu 10601PC Spectrophotometer (Tokyo, Japan) at 23 °C. Spectral binding titrations were performed to determine the dissociation constants (*K_d_*) for the substrate. Purified CYP102A1 mutant enzymes were diluted to 2 μM in 100 mM potassium phosphate buffer (pH 7.4) and divided into two glass cuvettes. Difference spectra in the range of 350–500 nm were recorded after each addition of substrate at the indicated concentration. Then, the absorbance differences were plotted against the added substrate concentration (0–100 µM). The *K_d_* values were estimated by fitting the absorbance difference data using nonlinear regression analysis using GraphPad Prism program (GraphPad Software, San Diego, CA, USA) [43].

## 3. Results

### 3.1. Hydroxylation of Phlorizin Catalyzed by CYP102A1 Mutants

**Figure 2:**
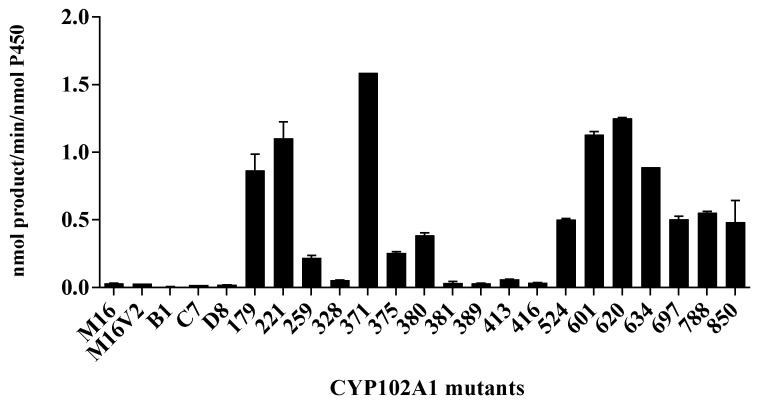
Phlorizin hydroxylation activity of CYP102A1 mutants. The reaction mixture contained phlorizin (200 μM) as a substrate in potassium phosphate buffer (100 mM, pH 7.4) and 0.40 μM of each CYP102A1 mutant. To initiate the reaction, NADPH-generating systems were added and the reaction was performed for 30 min at 37 °C.

**Figure 3:**
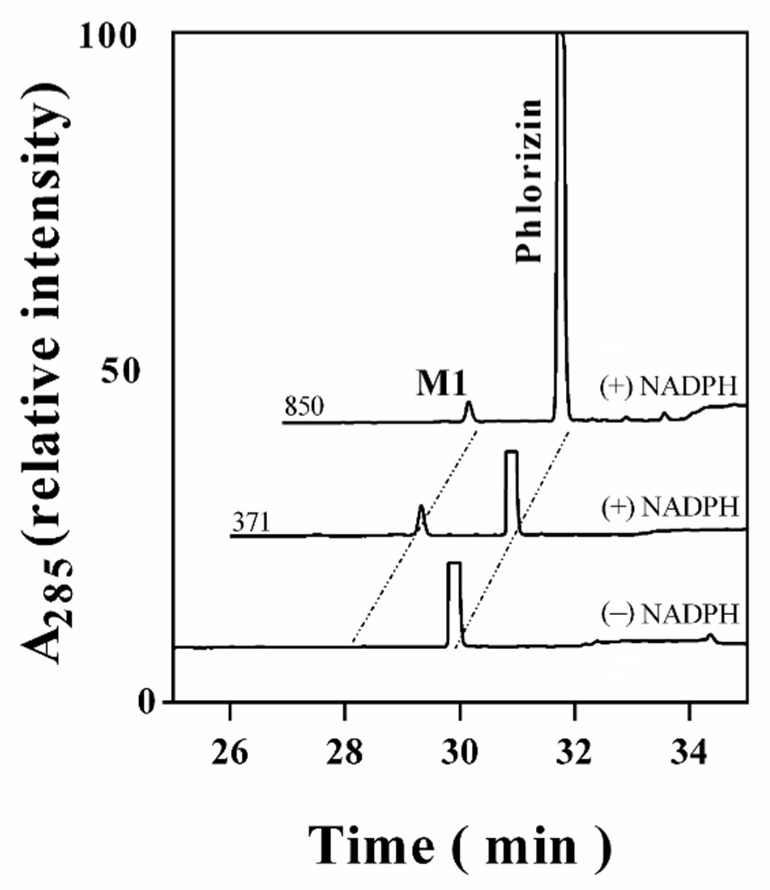
HPLC chromatogram of phlorizin and its products catalyzed by CYP102A1 mutants. The product peaks of reaction mixtures of HPLC traces were identified by comparing their retention times with that of phlorizin (*t_R_* = 30.1 min). The retention time of product M1 was 28.2 min.

**Figure 4:**
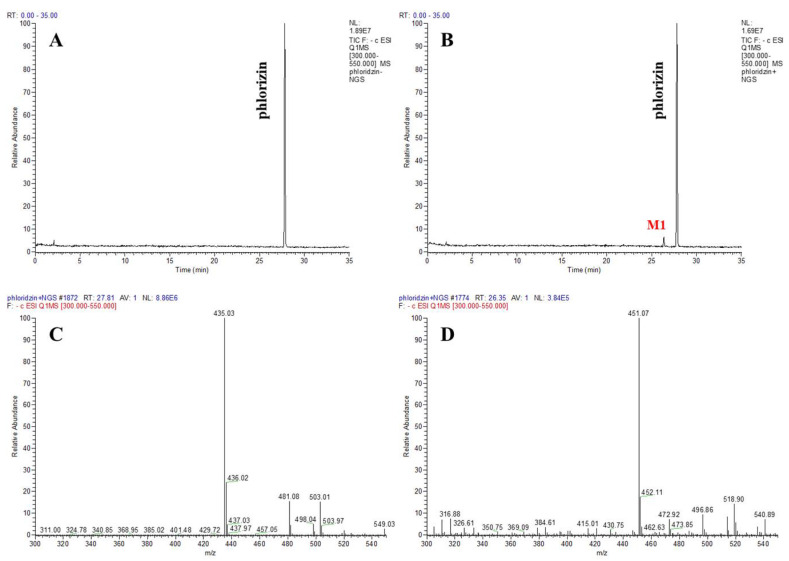
LC–MS analysis of phlorizin and its products produced by CYP102A1 M371 mutant. TIC of phlorizin without NADPH (**A**) and with NADPH (**B**). The MS spectra show that the protonated molecular ions ([M-H]^−^) of phlorizin (**C**) and its major product (M1) (**D**) were 435 and 451, respectively.

First, to determine the catalytic ability of CYP102A1 to hydroxylate phlorizin, the activity of the CYP102A1 WT and a panel of its mutants, which have high hydroxylation activities of polyphenol compounds, such as polydatin, naringin DC, and phloretin [7,38,40,41] toward phlorizin, was tested at the substrate concentration of 200 µM for 30 min at 37 °C (Figure 2). The CYP102A1 mutants for initial screening of highly active phlorizin hydroxylase were selected based on our previous works, which showed their high catalytic activities on a number of drugs and natural products (each mutated enzyme bears amino acid substitutions relative to WT CYP102A1, as summarized in Supplementary Table S1). The selection of mutants was based on their expression levels and phlorizin hydroxylation activity (Figure 2). Only one major metabolite was found from the incubation of phlorizin with all mutants (Figure 3). The mutants M371, M221, M620, and M850 show higher catalytic activity than M16V2 does (0.03 min^−1^). The activity of mutants M371 and M850 to make the major product was 5-fold higher than that of M16V2. The identities of the major products and substrate were confirmed by comparing the HPLC (Figure 3) and LC–MS analyses (Figure 4). To identify the product of phlorizin by M371, LC–MS analysis was performed with negative mode (Figure 4). The phlorizin ([M-H]^−^) has *m/z* 435, and the major product (M1) has *m/z* 451. This result indicates that M371 produces only one major product, a monohydroxylated product. We also found the optimal pH range of the phlorizin hydroxylation is pH 7.4–8 (Figure S1).

### 3.2. Characterizing a Major Product of Phlorizin by CYP102A1 and Subsequent β-Glucosidase

**Figure 5:**
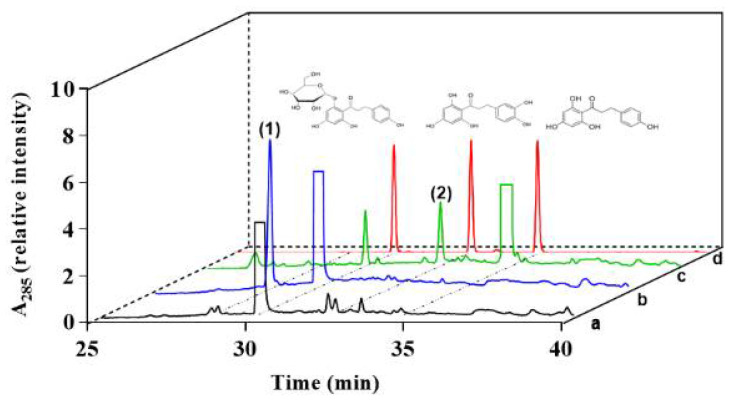
HPLC chromatograms of phlorizin and its major product produced by CYP102A1 M371. Phlorizin (0.2 mM) was treated with M371 in the absence (a) or presence (b) of NADPH. After the reaction was terminated, β-glucosidase was added (c). The peaks of the reaction mixtures were identified by comparing their retention times to those of the authentic compounds: (d) standard compounds of phlorizin (*t*_R_ = 30.1 min), 3-OH phloretin (*t*_R_ = 32.4 min), and phloretin (*t*_R_ = 34.6 min). Concentration of each compound was 10 μM. Peaks (1) and (2) represent 3-OH phlorizin and 3-OH phloretin, respectively.

First, phlorizin (200 μM) was incubated with M371 (0.4 μM) in the presence of NADPH to confirm the formation of the major product (Figure 5, line b). After the reaction was stopped with 2.4-fold volume of ice-cold ethyl acetate, the substrate and metabolites were extracted and dried. Subsequently, β-glucosidase (5 U) was added to the reaction mixture (*V_t_* = 0.25 mL) of phlorizin to determine the change in the product profile of M1 and substrate by treatment of β-glucosidase for 12 h. The M1 product and phlorizin were converted to 3-OH phloretin and phloretin, respectively (Figure 5, line c), while some portion of phlorizin remained. This result indicates that the 3-OH phlorizin is produced from phlorizin by treatment of CYP102A1.

**Figure 6:**
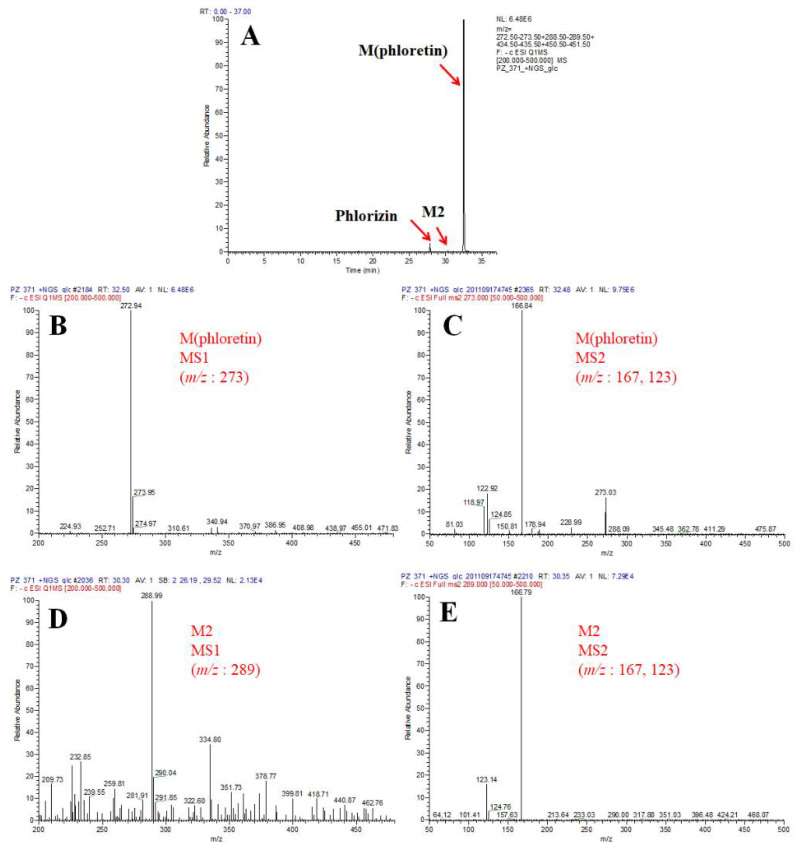
LC–MS/MS analysis of phlorizin and its products produced by treatment of CYP102A1 M371 and β-glucosidase. (**A**) TIC of phlorizin treated with M371 in the presence of NADPH, and subsequent treatment of β-glucosidase. (**B**,**C**) Each MS spectrum shows the protonated molecular ions (*m/z*: 273) of phloretin (**B**) and its daughter ions (MS2) with additional fragmentation (**C**). (**D**,**E**) The MS spectra show the protonated molecular ions (*m/z*: 289) of the product (M2) (**D**) and its daughter ions (MS2) with additional fragmentation (**E**).

To confirm the monohydroxylated product is 3-OH phlorizin, LC–MS/MS analysis was performed with the reaction mixture of M371-treated phlorizin and subsequent β-glucosidase treatment (Figure 6; Figures S2–S5). The total ion current (TIC) chromatogram of phlorizin treated with M371 and subsequent treatment of β-glucosidase showed three peaks of phlorizin (substrate), product M2, and product M (phloretin) (Figure 6A). The MS spectra show the protonated molecular ions (*m/z*: 273) of phloretin (M) and its daughter ions (M2) with additional fragmentation (MS2) (Figure 6B,C). The MS spectra show the protonated molecular ions (*m/z*: 289) of the product (M2) and its daughter ions (MS2) with additional fragmentation (Figure 6D,E). These results indicate that the product M2 is formed via initial hydroxylation and subsequent deglycosylation reactions of phlorizin.

To prove that the M2 is 3-OH phloretin, its fragmentation pattern (Figure 6E) was compared with that of 3-OH phloretin (Figure S6), which is formed from phloretin via the CYP102A1 M10 mutant [27]. The MS spectra of two samples that show the protonated molecular ions of the metabolite and its daughter ions with additional fragmentation were exactly matched. This result confirms that the M2 is 3-OH phloretin, which is formed via initial hydroxylation and subsequent deglycosylation reactions of phlorizin.

### 3.3. Kinetic Parameters and TTNs of Phlorizin Hydroxylation Catalyzed by CYP102A1 Mutants

Four CYP102A1 mutants were selected for further experiments to determine the kinetic parameters (*k_cat_* and *K_m_*) and total turnover numbers (TTNs, nmol product/nmol P450) of phlorizin 3-hydroxylation. Three mutants (M221, M371, and M850) had higher activities than that of M16V2, which was used as a template to make an enzyme library.

Table 1 shows the steady-state kinetics of a hydroxylated product formation from phlorizin by mutants M221, M371, M850, and M16V2. M221 has the highest *k_cat_* value of 1.1 min^−1^, which is 7-fold that of M16V2 (0.16 min^−1^). M850 and M371 also have increased *k*_cat_ values by 2.5- to 5.6-fold, respectively. However, the three mutants showed increased *K*_m_ values by 2.8–5.8 fold.

**Figure 7:**
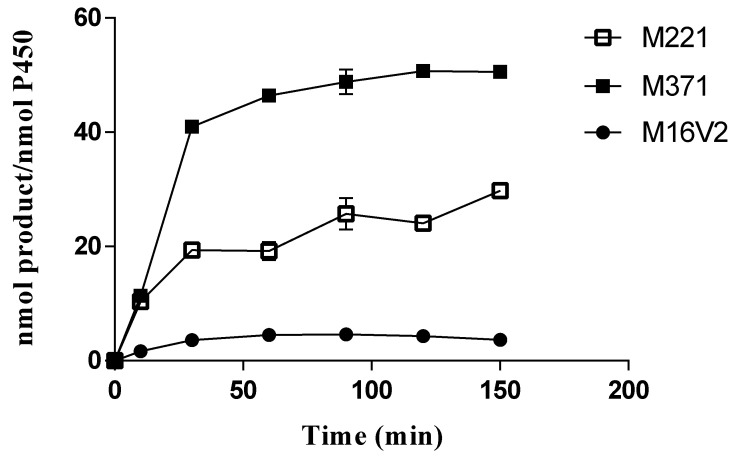
TTNs of phlorizin hydroxylation by CYP102A1 mutants. The reactions contained 0.2 mM of phlorizin substrate and 0.40 μM of CYP102A1 (M221, M371, and M16V2) in potassium phosphate buffer (100 mM, pH 7.4). To initiate the reaction, NADPH-generating systems were added to the reaction mixtures and incubated for 10, 30, 60, 90, 120, and 150 min at 37 °C.

Three mutants (M221, M371, and M16V2) were used to measure the TTNs of phlorizin hydroxylation (Figure 7). When the TTNs of the three mutants for the product formation were determined with 0.2 mM phlorizin during the incubation of 10, 30, 60, 90, 120, and 150 min, the overall range was 11 to 46 TTNs. M371 increased the product formation with incubation time up to 60 min and reached a plateau. However, the TTN of M221 was only 10–30, much lower than that of M371. Furthermore, the results showed that the production of 3-OH phlorizin by M221 and M371 reached the maximal at 60 min. Yields of the product formation by M371 and M221 were maximal of 10% and 4.8% at 150 min reaction.

### 3.4. Spectral Titration of Phlorizin toward CYP102A1 Mutants

**Figure 8:**
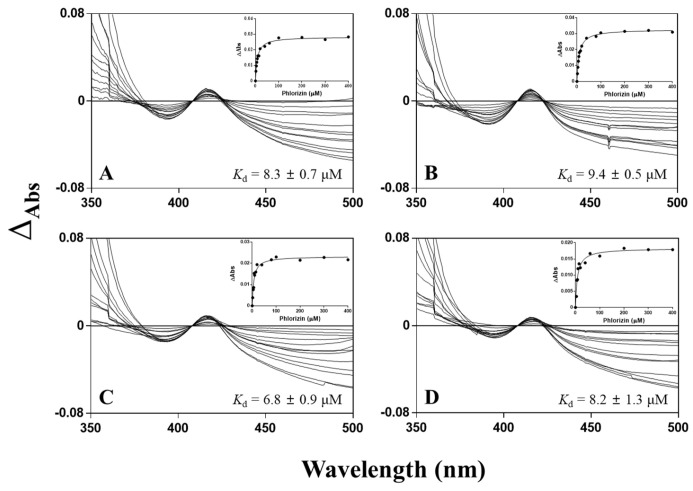
Spectral binding titration of CYP102A1 with phlorizin. The reactions contained 0–400 μM of phlorizin in potassium phosphate (100 mM, pH 7.4) and 2 μM P450 of M221 (**A**), M371 (**B**), M620 (**C**), and M850 (**D**). Insets show the plots of phlorizin absorption change calculation from different spectra obtained by subtraction of the staring spectrum from subsequently measured spectra collected at each phlorizin concentration in the titration.

To determine whether phlorizin can bind to the active site of CYP102A1, spectral binding titration was investigated with phlorizin (Figure 8). Phlorizin yielded a peak at 415 nm and a trough at 392 nm, which is consistent with type II binding of the ligand, indicating the substrate binds as a sixth ligand to the heme iron. In addition to the displacement of the coordinated water, the phlorizin itself serves as a ligand. The presence of increasing concentration of phlorizin enhanced the ΔA at lower phlorizin concentration and caused a continuous shift in trough position toward a lower wavelength. All tested mutants showed high binding affinity to phlorizin in the range of 6.8–9.4 μM. M620 bound to phlorizin with the highest affinity of a *K*_d_ value of 6.8 μM, whereas the other three mutants (M221, M371, and M850) apparently bound to phlorizin a little less tightly, with *K*_d_ values of 8.3, 9.4, and 8.2 μM, respectively.

## 4. Discussion

The most abundant polyphenols found in fruit, vegetables, and plants are natural flavonoids, and they are mostly in their glycosylated forms. However, their most biological activities, including antioxidants, are usually less pronounced in glycosides, but some specific bioactivities are enhanced [44]. The glycosylation of flavonoids has been found to increase their solubility and stability relative to flavonoid aglycones. Conjugation of low molecular weight flavonoid compounds by glycosylation is an efficient tool to increase water solubility, to improve stability, and thereby to increase or modify biological activity such as bioavailability. The development of a biocatalytic process is required to produce glycosides on a large scale [45]. Phlorizin belongs to DC compounds, a subclass of flavonoids.

Apple pomace is generally considered as an underutilized waste product of the apple processing industry. Apple pomace extracts can be used as a promising raw material for bioproduction of high value-added products via direct extraction of bioactive compounds such as polyphenols and pectin [46,47,48,49].

At present, phloretin and its glycosides are known to have beneficial biological activities for human health. These are versatile molecules with anticancer, anti-osteoclastogenic, anti-inflammatory, antibacterial, antifungal, antiviral, and estrogenic activities. They also can increase the fluidity of biological membranes and penetration of administered drugs into cells [50,51,52,53]. Furthermore, phloretin has attracted in the dermatological field for various applications in cosmetics and therapeutics [54].

Phlorizin is a glycoside of phloretin that has been widely investigated using animals, notably for its effects on various metabolic diseases, particularly diabetes [55,56]. Phlorizin is found in many parts including young shoots, leaves, roots, and bark in apple trees. In apple fruit, it is the most abundant in the seeds, with intermediate levels in the skin and the core, and the lowest level in the flesh [57]. The most well-known biological effect of phlorizin is its competitive inhibition of the glucose uptake via SGLT1 in the small intestine [58]. Phlorizin has been used as a pharmaceutical and tool for physiology research for over 150 years [59]. However, to the best of our knowledge, the production of phlorizin derivatives by chemical methods and their biological functions have never been reported previously.

Hydroxylation at the benzene ring is useful to produce a catechol moiety of phenolic compounds. Several P450s have been reported to mediate hydroxylation of phenolic compounds to produce catechol compounds. Bacterial CYP102A1 mutants were reported to exhibit hydroxylation activity toward resveratrol [60], polydatin [7], rhododendrol [61], and naringin DC [6] to produce catechol products. Interestingly, polydatin and naringin DC are glucosides and they could be regioselectively hydroxylated by CYP102A1. P450-catalyzed regioselective hydroxylation of natural glucosides at their phenol rings may be a highly efficient method to selectively change sensitive and complex natural product structures to generate new bioactive compounds. In this study, we have shown that bacterial CYP102A1 mutants could catalyze the hydroxylation reaction of phlorizin to produce 3-OH phlorizin, a catechol compound. Accordingly, an alternative way to chemical synthesis of the derivatives of phlorizin is to use CYP102A1 enzymes.

Moreover, 3-OH phlorizin is found in apples [25,62] and apple pomace [9]. It has exhibited antioxidant activities and cytotoxicity in cancer cell lines [62]. Enzymatic regioselective hydroxylation of phlorizin to make 3-OH phlorizin would be a highly favorable synthetic procedure. If 3-OH phlorizin can be synthesized from phlorizin enzymatically, its biological functions can be easily studied. Preparative scale reaction should be applicable using batch reaction process. However, whole-cell biocatalysis using recombinant CYP102A1 seems not to be applicable because 3-OH phlorizin was not produced when we attempted whole-cell biocatalysis with *Escherichia coli* cells expressing CYP102A1 (results not shown).

It is very interesting that CYP102A1 catalyzes the regioselective hydroxylation of both phlorizin and phloretin at the same C-3 position of the phenol ring. However, the hydrophilic sugar portion (i.e., glucose) might affect the regioselectivity of P450-catalyzed hydroxylation as it can constrain the orientation of the substrate to make a regioselectively-hydroxylated product. When the kinetic parameters of phlorizin hydroxylation and phloretin hydroxylation by selected CYP102A1 mutants were compared, all tested mutants showed higher *k_cat_* values for phloretin hydroxylation than those for phlorizin hydroxylation. In contrast, all the mutants show higher *K_m_* values for phlorizin than for phloretin. These results suggest that the CYP102A1 mutants have higher affinity for phloretin over phlorizin.

## 5. Conclusions

The glucoside of 3-OH phloretin would be a promising bioactive polyphenol agent, with increased bioavailability and water solubility compared with its aglycone. However, no procedures to make 3-OH phlorizin, a glucoside of 3-OH phloretin, by chemical and enzymatic methods are currently available. Biocatalytic regioselective hydroxylation of phlorizin to produce 3-OH phlorizin would be a highly favorable and eco-friendly synthetic procedure. Here, a biocatalytic strategy for the efficient synthesis of a potentially valuable hydroxylated product from phlorizin was developed via CYP102A1-catalyzed regioselective hydroxylation. The major product of phlorizin by CYP102A1 was found to be 3-OH phlorizin, which was confirmed by HPLC and LC–MS spectroscopy, in addition to enzymatic removal of its glucose moiety for comparison to 3-OH phloretin. Overall, in the present study, a panel of bacterial CYP102A1 mutants was developed to catalyze regioselective hydroxylation of phlorizin at the C-3 position to produce 3-OH phlorizin. Possible applications of CYP102A1 to regioselective C-hydroxylation of glycosides of polyphenols have been discussed.

## Figures and Tables

**Table 1:** Steady-state kinetics of the formation of 3-OH phlorizin from phlorizin using CYP102A1 mutants.

Enzymes	*k_cat_* (min^−1^)	*K_m_* (μM)	*k_cat_/K_m_* (min^−1^μM^−1^)
M221	1.1 ± 0.2	1540 ± 500	0.00071 ± 0.00027
M371	0.90 ± 0.2	979 ± 315	0.00091 ± 0.00036
M850	0.40 ± 0.02	747 ± 102	0.00053 ± 0.00008
M16V2	0.16 ± 0.01	267 ± 34	0.00059 ± 0.00008

## Data Availability

All data are presented in this manuscript.

## Supplementary Materials

The following are available online at https://www.mdpi.com/article/10.3390/antiox10081327/s1, Figure S1: pH-dependence of product formation of phlorizin catalyzed by CYP102A1 M371. Figure S2: LC–MS/MS analysis of phloretin standard. Figure S3: LC–MS/MS analysis of phlorizin standard. Figure S4: LC–MS/MS analysis of phlorizin with CYP102A1 M371 in the absence of NADPH. Figure S5: LC–MS/MS analysis of a major product of phlorizin produced by CYP102A1 M371 in the presence of NADPH. Figure S6: LC–MS/MS analysis of a major product of phloretin produced by CYP102A1 M10 in the presence of NADPH. Figure S7: Standard curves of internal standard, phloretin, and phlorizin. Table S1: The amino acid sequence of M16V2 and CYP102A1 mutants.
