# Supplementary material for: Enzymatic Production of 3-OH Phlorizin, a Possible Bioactive Polyphenol from Apples, by Bacillus megaterium CYP102A1 via Regioselective Hydroxylation
Source: Antioxidants (Basel). 2021 Aug 23;10(8):1327. doi: 10.3390/antiox10081327 (PMC8406095; doi:10.3390/antiox10081327)
Supplement: Supplementary file 1 [file antioxidants-10-01327-s001.zip › antioxidants-1338207-SI.pdf]

antioxidants

MDPI

Supporting Information

Enzymatic Production of 3-OH Phlorizin, a Possible Bioactive Polyphenol from Apples, by *Bacillus megaterium* CYP102A1 via Regioselective Hydroxylation

Ngoc Anh Nguyen [1], Ngoc Tan Cao [1], Thi Huong Ha Nguyen [1], Jung-Hwan Ji [1], Gun Su Cha [2], Hyung-Sik Kang [1,3] and Chul-Ho Yun [1,3,*]

[1] School of Biological Sciences and Biotechnology, Graduate School, Chonnam National University, Yongbong-ro 77, Gwangju 61186, Korea; nguyenanh@jnu.ac.kr (N.A.N.); 187529@jnu.ac.kr (N.T.C.); huongha0207@gmail.com (T.H.H.N.); 216704@jnu.ac.kr (J.-H.J.); kanghs@jnu.ac.kr (H.-S.K.)
[2] Namhae Garlic Research Institute, 2465-8 Namhaedaero, Gyeongsangnamdo 52430, Korea; gscha450@gmail.com
[3] School of Biological Sciences and Technology, Chonnam National University, Yongbong-ro 77, Gwangju 61186, Korea
* Correspondence: chyun@jnu.ac.kr; Tel. +82-62-530-2194

Citation: Nguyen, N.A.; Cao, N.T.; Nguyen, T.H.H.; Ji, J.-H.; Cha, G.S.; Kang, H.-S.; Yun, C.-H. Enzymatic Production of 3-OH Phlorizin, a Possible Bioactive Polyphenol from Apples, by *Bacillus megaterium* CYP102A1 via Regioselective Hydroxylation. *Antioxidants* **2021**, *10*, 1327. https://doi.org/10.3390/antiox10081327

Academic Editor: David Arráez-Román

Received: 28 July 2021
Accepted: 20 August 2021
Published: 23 August 2021

Publisher's Note: MDPI stays neutral with regard to jurisdictional claims in published maps and institutional affiliations.

Copyright: © 2021 by the authors. Licensee MDPI, Basel, Switzerland. This article is an open access article distributed under the terms and conditions of the Creative Commons Attribution (CC BY) license (http://creativecommons.org/licenses/by/4.0/).

Table S1. The amino acid sequence of M16V2 and CYP102A1 mutants

[a]M16	R47L/F81I/F87V/E143G/L188Q/E267V
[a]M16V2	R47L/F81I/F87V/E143G/L188Q/E267V/A474V/E558D/T664A/P675L/A678E/E687A/A741G/K813E/R825S/R836H/E870N/I881V/E887G/P894S/S954N/M967V/Q981R/A1008D/H1021Y/Q1022E[c]
	Mutants B1-M850 contain underlined 20 mutated amino acids of V2 reductase domain of M16V2
[a]B1	R47L/F81I/F87V/E143G/L188Q/E267V/D351G
[a]C7	R47L/K59N/F81I/F87V/E143G/L188Q/G240E/E267V/S383R
[a]D8	R47L/F81I/F87V/E143G/L188Q/E267V/Y334C/A335V/D369G
[b]M179	R47L/F81I/F87V/E143G/L188Q/N213S/E267V
[b]M221	F11Y/R47L/F81I/F87V/E143G/L188Q/E267V/H408R
[b]M225	D23G/R47L/F81I/F87V/E143G/L188Q/E267V/E409D
[b]M250	R47L/F81I/F87V/E143G/L188Q/M212V/E267V/K309N
[b]M259	R47L/F81I/F87V/E143G/T149S/L188Q/E267V/S270G
[b]M301	R47L/F81I/F87V/S108C/E143G/T149S/L188Q/E267V
[b]M306	R47L/F81I/F87V/M112T/E143G/L188Q/E267V/M417T
[b]M326	R47L/F81I/F87V/K113E/E143G/T152S/L188Q/F261L/E267V
[b]M328	R47L/F81I/F87V/E143G/K187E/L188Q/V211M/E267A/S274T
[b]M371	D23G/R47L/F81I/F87V/F107L/D136G/E143G/L188Q/E267V
[b]M375	R47L/F81I/F87V/S106C/Q109R/E143G/L188Q/E267V/D338E
[b]M380	R47L/F81I/F87V/L103F/D136G/E143G/N159S/L188Q/E267V
[b]M381	R47L/F81I/F87V/W96R/S106R/E143G/L188Q/E267V/I401V
[b]M389	D23G/R47L/F81I/D84N/F87V/E143G/G154S/M185V/L188Q/E267V
[b]M413	R47L/F81I/F87V/Q128R/E143G/L188Q/E267V/L287S/K309R/S383C
[b]M416	R47L/S72C/F81I/F87V/S108G/E143G/F158L/L188Q/M212V/E267V/E344D
[b]M524	F42L/R47L/F81I/F87V/E143G/L188Q/E267V
[b]M601	R47L/F81I/F87V/E143G/L150F/L188Q/E267V
[b]M620	R47L/F81I/F87V/Q109L/E143G/L188Q/D199V/E267V

[b]M634	T1S/R47L/F81I/F87V/E143G/T146A/L188Q/E267V
[b]M697	R47L/F81I/F87V/E143G/L188Q/E267V/K312N/D370E
[b]M788	R47L/F81I/F87V/K113N/E143G/L188Q/E267V/N319D/L347I/S383R
[b]M850	F11Y/R47L/D68G/F81I/F87V/E143G/L188Q/E267V/H408R

[a]Chimera M16V2 and its mutants were reported previously [1,2]. Twenty six mutants (B1~M850) of chimera M16V2 were obtained by error-prone PCR of heme domain of the chimera M16V2, generation and expression of DNA library, and HTS system [2,3].

[b]Underlined: amino acid from variant V2 [1].

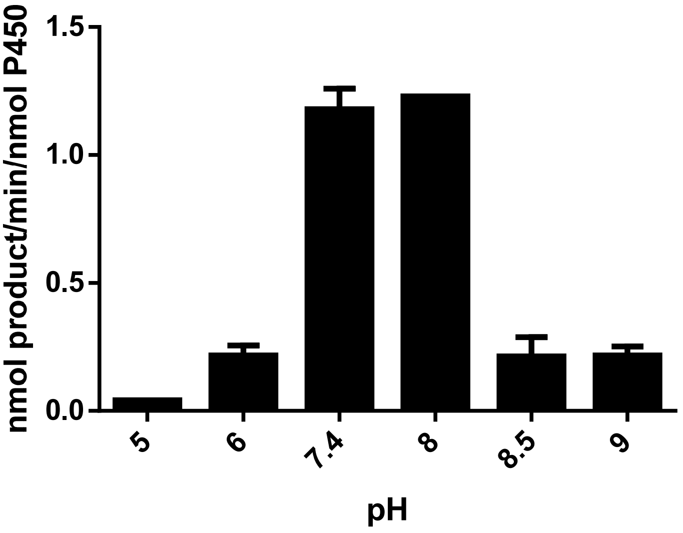

Figure S1. pH-dependence of product formation of phlorizin catalyzed by CYP102A1 M371. Effect of pH on catalytic activity of M371 for the formation of phlorizin product was done using 200 μM phlorizin substrate in different pH for 1 h at 37 °C. 100 mM of potassium phosphate buffer and sodium acetate buffer were used for pH 5 and pH 6-9, respectively.

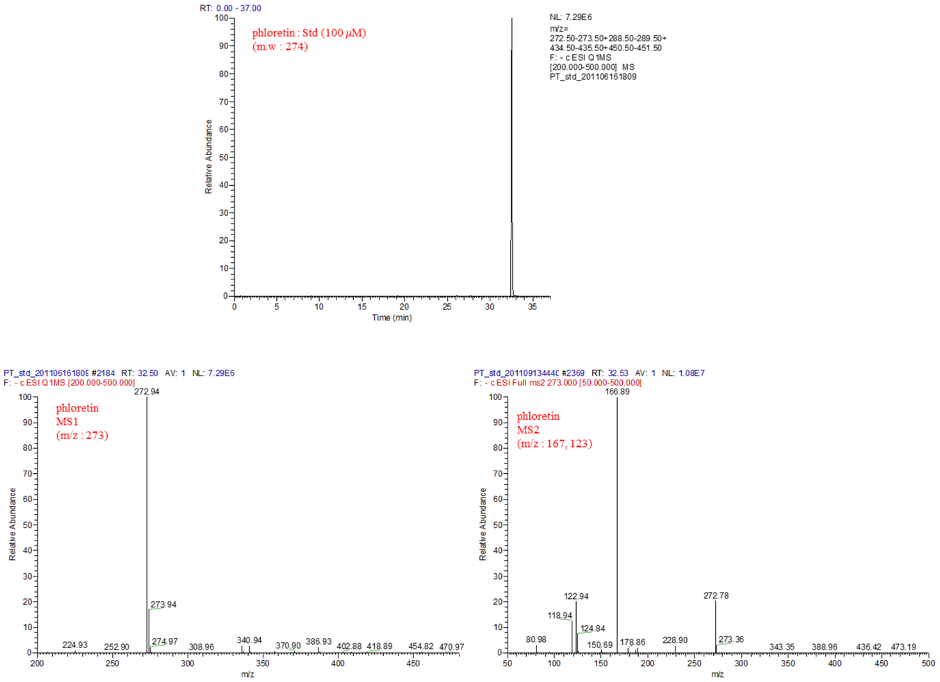

Figure S2. LC-MS/MS analysis of phloretin standard. TIC of phloretin (top), the MS spectra of the protonated molecular ions of phloretin (MS1) (bottom, left), and its daughter ions (MS2) with additional fragmentation (bottom, right) were shown.

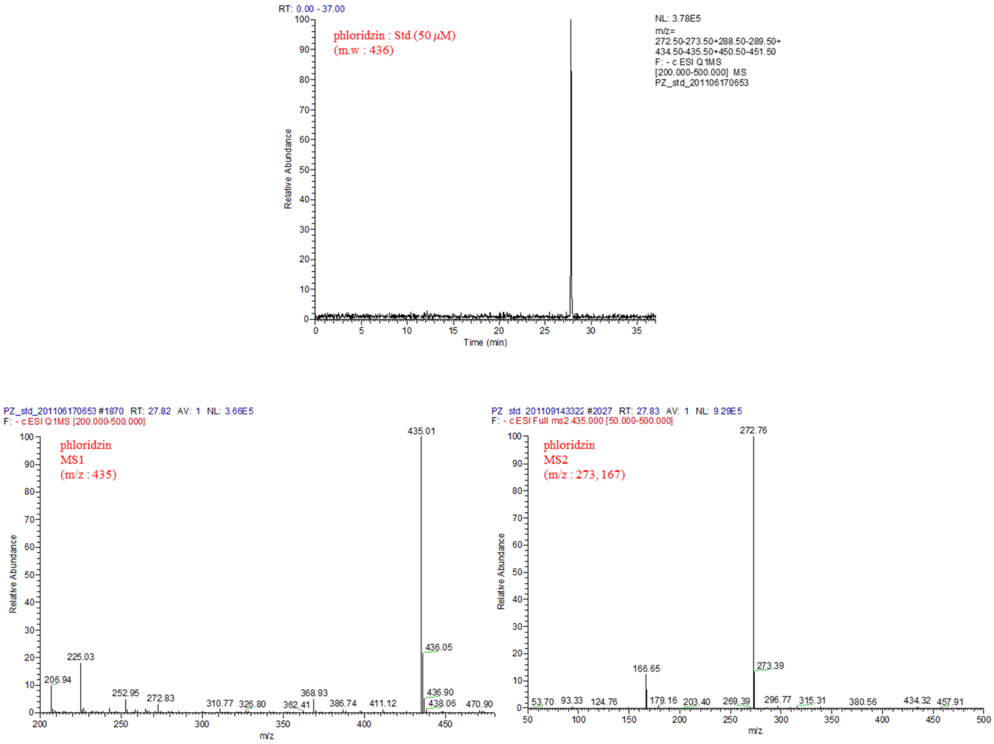

Figure S3. LC-MS/MS analysis of phlorizin standard. TIC of phlorizin (top), the MS spectra of the protonated molecular ions of phlorizin (MS1) (bottom, left), and its daughter ions (MS2) with additional fragmentation (bottom, right) were shown.

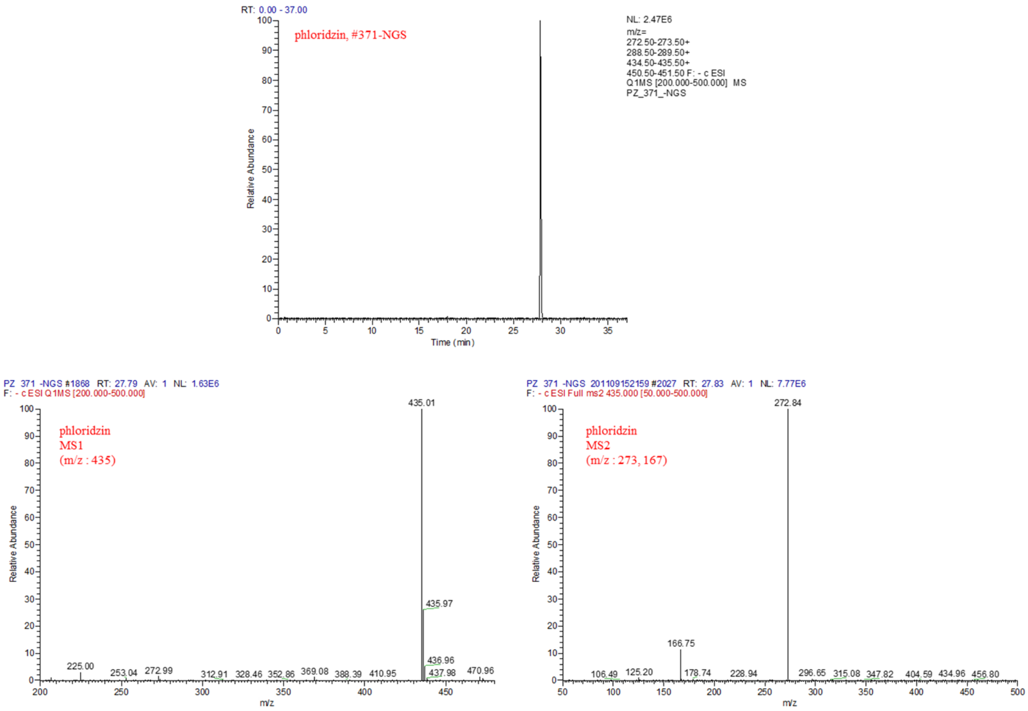

Figure S4: LC-MS/MS analysis of phlorizin with CYP102A1 M371 in the absence of NADPH. TIC of phlorizin (top), the MS spectra of the protonated molecular ions of phlorizin (MS1) (bottom, left), and its daughter ions (MS2) with additional fragmentation (bottom, right) were shown.

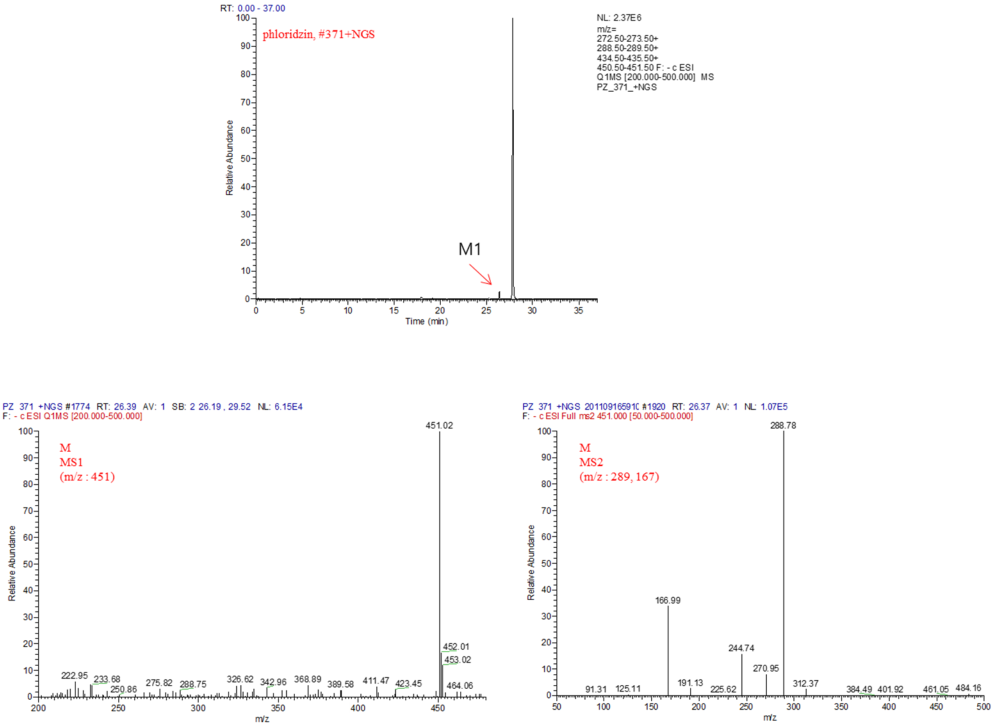

Figure S5. LC-MS/MS analysis of a major product of phlorizin produced by CYP102A1 M371 in the presence of NADPH. TIC of phlorizin and its major product (M1) (top), the MS spectra of the protonated molecular ions of the product (MS1) (bottom, left), and its daughter ions (MS2) with additional fragmentation (bottom, right) were shown.

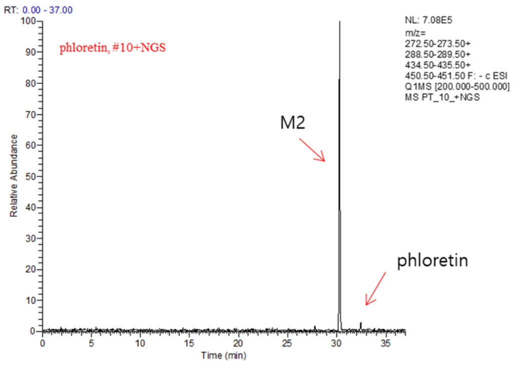

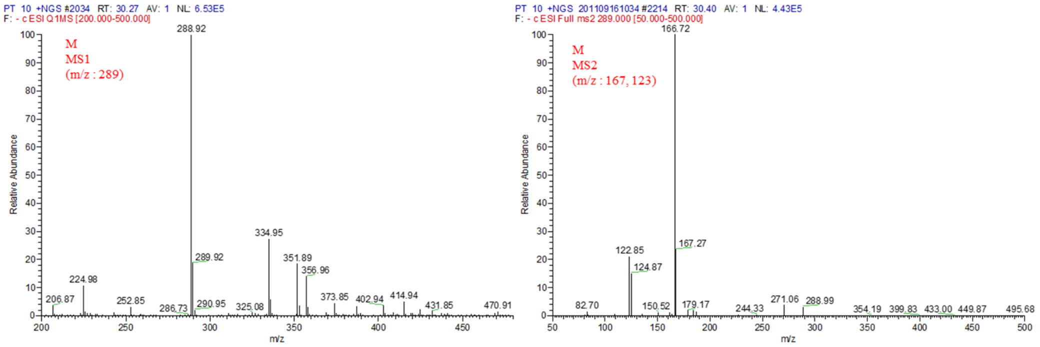

Figure S6. LC-MS/MS analysis of a major product of phloretin produced by CYP102A1 M10 in the presence of NADPH. TIC of phloretin and its major product (M2) (top), the MS spectra of the protonated molecular ions of the product (MS1) (bottom, left), and its daughter ions (MS2) with additional fragmentation (bottom, right) were shown.

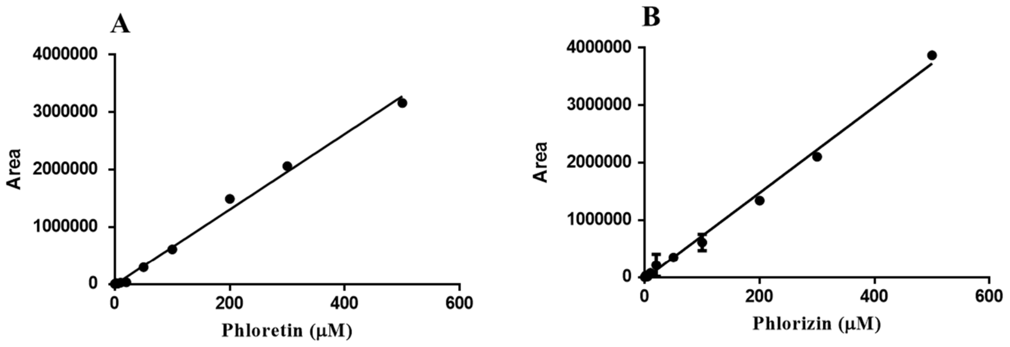

Phloretin- Slope	6576 ± 109
Y- intercept	-8837 ± 19737
R square	0.9940
Equation	Y=6576*X - 8837

Phlorizin- Slope	7504 ± 136
Y- intercept	-25157 ± 24648
R square	0.9928
Equation	Y=7504*X - 25157

Figure S7. Standard curves of internal standard, phloretin, and phlorizin. (A) Twelve different concentrations of phloretin were used at 0.2, 0.5, 2, 5, 10, 20, 30, 50, 100, 200, 300, and 500 µM. (B) Twelve different concentrations of phlorizin were used at 0.2, 0.5, 2, 5, 10, 20, 30, 50, 100, 200, 300, and 500 µM .

References for Supporting Information

1. Kang, J.-Y.; Kim, S.-Y.; Kim, D.; Kim, D.-H.; Shin, S.-M.; Park, S.-H.; Kim, K.-H.; Jung, H.-C.; Pan, J.-G.; Joung, Y.H.; et al. Characterization of Diverse Natural Variants of CYP102A1 Found within a Species of Bacillus Megaterium. *AMB Express* **2011**, *1*, 1, doi:10.1186/2191-0855-1-1.
2. Kang, J.-Y.; Ryu, S.H.; Park, S.-H.; Cha, G.S.; Kim, D.-H.; Kim, K.-H.; Hong, A.W.; Ahn, T.; Pan, J.-G.; Joung, Y.H.; et al. Chimeric Cytochromes P450 Engineered by Domain Swapping and Random Mutagenesis for Producing Human Metabolites of Drugs. *Biotechnol. Bioeng.* **2014**, *111*, 1313–1322, doi:10.1002/bit.25202.
3. Nguyen, T.H.H.; Woo, S.-M.; Nguyen, N.A.; Cha, G.-S.; Yeom, S.-J.; Kang, H.-S.; Yun, C.-H. Regioselective Hydroxylation of Naringin Dihydrochalcone to Produce Neoeriocitrin Dihydrochalcone by CYP102A1 (BM3) Mutants. *Catalysts* **2020**, *10*, 823, doi:10.3390/catal10080823.
